# Effects of HIV infection, antiretroviral therapy, and immune status on the speed of information processing and complex motor functions in adult Cameroonians

**DOI:** 10.1038/s41598-020-70981-4

**Published:** 2020-08-20

**Authors:** Georgette D. Kanmogne, Julius Y. Fonsah, Anya Umlauf, Jacob Moul, Roland F. Doh, Anne M. Kengne, Bin Tang, Claude T. Tagny, Emilienne Nchindap, Léopoldine Kenmogne, Donald Franklin, Dora M. Njamnshi, Callixte T. Kuate, Dora Mbanya, Alfred K. Njamnshi, Robert K. Heaton

**Affiliations:** 1 Vice-Chair for Resource Allocation and Faculty Development, Department of Pharmacology and Experimental Neuroscience, College of Medicine, University of Nebraska Medical Center, Omaha, NE 68198-5800 USA; 2 Faculty of Medicine and Biomedical Sciences, University of Yaoundé I, Yaoundé, Cameroon; 3 Department of Neurology, Yaoundé Central Hospital/Brain Research Africa Initiative (BRAIN), Yaoundé, Cameroon; 4 Department of Psychiatry, University of California San Diego, 9500 Gilman Drive, La Jolla, CA 92093 USA; 5 Yaoundé University Teaching Hospital, Yaoundé, Cameroon; 6 HIV-Day Care Service, Yaoundé Central Hospital, Yaoundé, Cameroon; 7 Department of Neurology, Laquintinie Hospital, Douala, Cameroon

**Keywords:** HIV infections, Central nervous system infections, Human behaviour, Neurological disorders

## Abstract

HIV-associated neurocognitive deficits include impaired speed-of-information processing (SIP) and motor functions. There is lack of Cameroonian adult norms for assessing SIP or motor functions. This study of 683 Cameroonians (320 HIV+, 363 HIV−) establishes demographically-adjusted norms for six SIP [Wechsler-Adult-Intelligence-Scale (WAIS)-III Digit Symbol (WAIS-IIIDS) and Symbol Search (WAIS-IIISS), Stroop Color-Naming, Stroop Word-Reading, Trail-Making Test-A (TMT-A), Color Trails-1 (CTT1)], and two motor function [Grooved Pegboard-dominant (GP-DH) and non-dominant (GP-NDH) hands] tests. We assessed viral effects on SIP and motor functions. HIV-infected persons had significantly lower (worse) T scores on GP-DH, WAIS-IIIDS, Stroop Word-Reading, TMT-A; lower motor and SIP summary T scores. Significantly higher proportion of cases (20.7%) than controls (10.3%) had impaired SIP. Male cases had better T scores than female cases on GP-NDH, WAIS-IIIDS, WAIS-IIISS, TMT-A, CTT1; better SIP summary T scores. Antiretroviral therapy (ART) was associated with significantly better T scores on GP-NDH, WAIS-IIIDS, Stroop Color-Naming; better motor and SIP summary T scores. Cases with higher CD4 had better T scores on WAIS-IIIDS, TMT-A, CTT1; better SIP summary T scores. Overall, we demonstrate that HIV infection in Cameroon is associated with deficits in SIP and motor functions; ART and higher CD4 are associated with better cognitive performance. We provide SIP and psychomotor functions normative standards, which will be useful for neurobehavioral studies in Cameroon of diseases affecting the brain.

## Introduction

Diseases that affect the central nervous system (CNS) often result in impaired cognition. This is the case for HIV/AIDS, where in the early stages of infection, the virus induces blood–brain barrier injury, enters the CNS, and productively infects resident macrophages and glial cells^1,2^. This infection of CNS cells, production and release of HIV virions and viral proteins into the brain, as well as subsequent increased inflammation and oxidative stress, can cause neuronal injury and death, and result in behavioral, motor and cognitive abnormalities termed HIV-associated neurocognitive disorders (HAND)^2–4^. Antiretroviral therapy (ART) is associated with improved cognition^5–8^ and ART failure correlates with poor performance on tests of neurocognitive function^9^. Cross-sectional and longitudinal studies have shown that the odds of neurocognitive impairment increased in subjects with high plasma viral loads (VL)^10^ and that such impairment is associated with poor health-related quality of life^11^. Although the prevalence of HIV-associated dementia (HAD), the most severe form of HAND, has markedly decreased in the current ART era^5,12,13^, milder forms of HAND [asymptomatic neurocognitive impairment and mild neurocognitive disorders] and overall neurocognitive impairment still occur in up to 50% of HIV-infected persons^13–17^.

HAND involves impairments in several cognitive domains, including concentration and mental processing, memory and motor domains^14,15,18^. In fact, HIV infection is associated with slower speed of cognitive processing^19,20^ and HIV-induced deficits in speed of information processing (SIP) may be associated with other cognitive abnormalities, as there is evidence that the SIP affects performance on other cognitive domains such as learning, memory and executive function^21^. Decline in motor functions is also common in HIV-infected individuals^22–24^, is associated with cortical gray matter atrophy^25^, and is a predictor of subsequent cognitive impairments^24^.

HIV/AIDS epidemiology is characterized by a high clade diversity and differential geographic distribution based on viral subtypes^26,27^. There is evidence that the frequency of neurocognitive impairments among infected subjects varies based on HIV subtypes^13,28–31^ but current understanding of HIV neuropathology and HAND mostly comes from studies of Western populations infected with HIV-1 clade-B^14,15,17^. Over two-thirds of the 38 million people living with HIV/AIDS (PLWH) are in Sub-Saharan Africa (SSA) and are mostly infected with different (non-B) HIV clades^32^. Thus, it is important to investigate the prevalence and risk factors of HAND in these populations. The neuropsychological (NP) measures used to assess cognitive abilities and diagnose neurocognitive impairments in humans are influenced by demographic factors such as age, education, ethnicity, and sex, as well as by cultural and ethnic backgrounds^33,35^. Therefore, population-appropriate normative standards for these NP measures are critical to accurately assess the neurobehavioral effects of HIV infection.

Cameroon is a SSA country with a 3.8% HIV adult prevalence (total population of 25 million inhabitants)^35^ and HIV/AIDS epidemiology characterized by a high viral genetic diversity^36–38^. Given the absence of adult norms for assessing SIP or motor functions in the Cameroon population, the objective of the current study was to develop demographically-adjusted normative standards for six commonly used NP tests of SIP [Wechsler Adult Intelligence Scale (WAIS)-III Digit Symbol and Symbol Search (WAIS-IIIDS and WAIS-IIISS)^39^, Stroop Color Naming and Word Reading speed^40^, Trail Making Test Part-A Time (TMT-A)^41^, and Color Trails-1 Time (CTT1)^42^], and two speeded measures of fine motor function [Grooved Pegboard Test dominant hand and non-dominant hand trials (GP-DH and GP-NDH)]^43^. Because HIV infection and viral factors affect neurocognitive performance, our secondary objectives were to assess the effects of HIV infection, immune function, VL, viral genotype and ART on subjects’ performance on these tests of SIP and complex motor functions.

## Results

### Participants and laboratory characteristics

Data from 683 subjects (363 HIV− controls and 320 HIV+ cases) were used in this study. Demographic description for each group is given in Table 1. The participants ranged in age from 18 to 64 years, with majority being females (71.3%) and the number of years of formal education ranged from 0 to 21 years. Controls were on average younger, more educated, and had a higher proportion of males (Table 1). The HIV+ cases had a median CD4 cell count of 405 cells/μl, the majority were on ART (53.6%) and had controlled viremia (57.2% had undetectable VLs) (Table 1).

**Table 1:** Demographic and clinical characteristics by HIV status.

Characteristics	HIV−		HIV+		P value
	N^a^	Mean (SD) or N (%)	N^a^	Mean (SD), Median [IQR], or N (%)	
Demographics					
Age (years)	363	34.3 (10.6)	320	37.8 (9.4)	< 0.001
Age range [IQR] (years)		18–64 [26, 42]		18–60 [31, 45]	
Education (years)	363	12.4 (4.2)	320	9.6 (3.7)	< 0.001
Formal education range [IQR] (years)		0–21 [9, 16]		2–20 [6, 12]	
Male, N (%)	363	125 (34.4%)	320	71 (22.2%)	< 0.001
HIV disease					
CD4	–	–	289	405 [246, 574]	–
Viral load, N (%)	–	–	290		
Undetectable	–	–	–	166 (57.2%)	–
Detectable	–	–	–	124 (42.8%)	–
Log10 viral load (among subjects with detectable VL)	–	–	124	4.61 (1.30)	–
HIV-1 CRF02_AG subtypes	–	–	–	89 (58.2%)	–
Non-CRF02_AG subtypes	–	–	–	64 (41.8%)	–
ART status, N (%)			319		
ART	–	–	–	171 (53.6%)	–
Naïve	–	–	–	142 (44.5%)	–
Not current	–	–	–	5 (1.6%)	–
Other (1 ZIDOVIR in pregnancy only, and 1 Vanhivax)	–	–	–	1 (0.3%)	–

Values are Mean (SD), Median [IQR], or N (%).

Student’s t test was applied for continuous variables, and Fisher’s exact test for categorical variables.

*SD* standard deviation, *IQR* interquartile range.

^a^Total number of participants with available data for the corresponding variable.

### Conversion of raw scores to standardized scaled scores

Table 2 shows details on scaled scores and corresponding raw scores for tests assessing motor functions [GP-DH and GP-NDH (time)] and SIP [WAIS-IIIDS (total scores); WAIS-IIISS (total scores); Stroop Color Naming (total correct), Stroop Word Reading (total correct), TMT-A (time), and CTT1 (time)]. Table 3 shows the equations used for regression-based analyses and calculation of demographically-corrected T scores for tests of SIP and motor functions.

**Table 2:** Conversion of the raw scores to scaled scores for tests assessing motor and speed of information processing domains.

Scaled score	Motor		Speed of information processing						
	Grooved pegboard dominant hand time (s)	Grooved pegboard non-dominant hand time (s)	WAIS-III digit symbol total	WAIS-III symbol search total	Stroop color	Stroop words	Trail making A time (s)	Color trails 1 time (s)	Scaled score
1	355–360	362–375	–	− 60 to − 17	0–7	0–15	242–255	304–345	1
2	251–354	307–361	0–4	− 16 to − 2	8–24	16–25	200–241	215–303	2
3	173–250	260–306	5–10	− 1 to 0	25–28	26–38	141–199	161–214	3
4	134–172	181–259	11–18	1 to 4	29–32	39–40	122–140	138–160	4
5	117–133	129–180	19–23	5 to 7	33–38	41–53	98–121	114–137	5
6	99–116	118–128	24–29	8 to 10	39–43	54–63	85–97	89–113	6
7	88–98	105–117	30–34	11 to 13	44–47	64–68	71–84	76–88	7
8	82–87	95–104	35–40	14 to 16	48–52	69–74	62–70	64–75	8
9	77–81	89–94	41–47	17 to 19	53–57	75–80	54–61	57–63	9
10	72–76	83–88	48–52	20 to 23	58–61	81–86	47–53	51–56	10
11	68–71	79–82	53–59	24 to 26	62–68	87–93	42–46	46–50	11
12	65–67	76–78	60–65	27 to 28	69–72	94–98	36–41	41–45	12
13	61–64	71–75	66–71	29 to 31	73–78	99–101	32–35	36–40	13
14	59–60	67–70	72–76	32 to 34	79–81	102–110	28–31	33–35	14
15	55–58	64–66	77–81	35 to 38	82–88	111–118	24–27	29–32	15
16	53–54	62–63	82–86	39	89–93	119–126	20–23	25–28	16
17	47–52	59–61	87–93	40 to 46	94–98	127–130	18–19	21–24	17
18	10–46	52–58	94–100	47 to 58	99–101	131–133	14–17	0–20	18
19	0–9	0–51	101–133	59 to 60	102–133	–	0–13	–	19

*S* seconds.

**Table 3:** T score calculation formulas based on scaled scores for tests assessing motor and speed of information processing domains.

Test	Formula
**Motor domain**	
Grooved Pegboard–dominant hand	50 + 10 × [(scaled score) − (9.9524 + 2.1445 × ((edu + 1)/10) − 8.0012 × (age/100) − 0.2450 × male)]/2.6106
Grooved Pegboard–non-dominant hand	50 + 10 × [(scaled score) − (11.1141 + 1.7112 × ((edu + 1)/10) − 9.4319 × (age/100) − 0.4858 × male)]/2.6828
**SIP domain**	
WAIS-III digit symbol total	50 + 10 × [(scaled score) − (8.3622 + 3.9066 × ((edu + 1)/10) − 9.8019 × (age/100) − 0.5678 × male)]/2.1019
WAIS-III symbol search	50 + 10 × [(scaled score) − (3.9602 + 3.5142 × ((edu + 1)/10) + 0.1238 × (age/100)^−2^ − 0.0233 × male)]/2.4067
Stroop color total	50 + 10 × [(scaled score) − (9.2469 + 2.1281 × ((edu + 1)/10)^3^ − 2.4993 × log((edu + 1)/10) × ((edu + 1)/10)^3^ − 5.9936 × (age/100) − 0.4210 × male)]/2.6168
Stroop words total	50 + 10 × [(scaled score) − (7.9642 + 2.4560 × ((edu + 1)/10)^3^ − 2.8511 × log((edu + 1)/10) × ((edu + 1)/10)^3^ − 3.9559 × (age/100) − 0.1437 × male)]/2.5853
Trail making A time	50 + 10 × [(scaled score) − (9.1490 + 2.6381 × ((edu + 1)/10) − 7.5250 × (age/100) − 0.3085 × male)]/2.5746
Color trails 1 time	50 + 10 × [(scaled score) − (8.6428 + 2.7874 × ((edu + 1)/10) − 6.8461 × (age/100) − 0.1505 × male)]/2.5560

*Edu* education, *male* 1 for male, 0 for female, *SIP* speed of information processing, *WAIS-III* Wechsler Adult Intelligence Scale-*III.*

### Effects of age, education, and gender on tests of complex motor function raw scores and standardized scores

Analysis of controls’ raw scores showed older age and lower level of education being associated with worse performance on the GP-DH or GP-NDH tests (Ps < 0.001), but no gender effect. As expected, the controls’ T scores showed no effect of age, education, or gender on subjects’ performance on these tests. There was no effect of age or education on cases’ T scores for the GP-DH or GP-NDH tests, or the overall motor summary T score. However, a gender effect (males’ T scores better than females’) was observed on cases’ GP-NDH T scores [coefficient (C): 2.87, 95% confidence interval (CI): 0.11, 5.62; P = 0.041; Adj. P = 0.066]; but gender did not influence cases’ performance on the GP-DH or the overall motor summary score.

### Effects of age, education, and gender on tests of SIP raw scores and standardized scores

Analyses of controls’ raw scores showed significant effect of age and education (younger age and higher level of education associated with better performance) on WAIS-IIIDS (total scores); WAIS-IIISS (total scores); Stroop Color Naming, Stroop Word Reading, TMT-A (time), and CTT1 (time). There was no significant effect of gender on tests of SIP among controls, with the exception of WAIS-IIISS raw scores that showed significantly better performance by males compared to females (C: 2.13; 95% CI 0.07, 4.19; P = 0.043; Adj. P = 0.34). Corrected T scores showed no age, education, or gender effects on tests of SIP for HIV− controls.

Although normal effects of demographics were fully controlled in the HIV− controls’ T scores, age influenced T scores of cases (older age associated with worse T scores) on WAIS-IIIDS (C: 0.14, 95% CI 0.03, 0.25; P = 0.014; Adj. P = 0.056); WAIS-IIISS (C: 0.14, 95% CI 0.01, 0.27; P = 0.038; Adj. P = 0.10), and Stroop Color Naming (C: 0.24, 95% CI 0.11, 0.37; P < 0.001; Adj. P = 0.003), as well as the SIP summary T scores (C: 0.11, 95% CI 0.03, 0.19; P = 0.009; Adj. P = 0.018). There was no gender effect on cases’ Stroop Color Naming or Stroop Word Reading T scores. However, analyses of cases showed gender effects (females scoring significantly lower than males) on T scores for WAIS-IIIDS (C: 3.78, 95% CI 1.28, 6.28; P = 0.003; Adj. P = 0.012), WAIS-IIISS (C: 3.33, 95% CI 0.43, 6.23; P = 0.024; Adj. P = 0.048), TMT-A (C: 6.81, 95% CI 4.12, 9.51; P < 0.001; Adj. P < 0.001), and CTT1 (C: 2.93, 95% CI 0.42, 5.44; P = 0.022; Adj. P = 0.048); there also were gender effects (again, females scoring lower than males) on the overall SIP summary T scores (C: 2.82, 95% CI 0.97, 4.67; P = 0.003; Adj. P = 0.018). This means that, even when Cameroonian “normal” female disadvantages on these tests are controlled, female cases showed evidence of additional gender disadvantages.

### Effects of HIV infection on complex motor function and SIP

#### Motor

Comparative analyses of cases and controls showed no group difference in the GP-NDH T scores, but cases had significantly worse T scores on GP-DH (P = 0.018; Adj. P = 0.036) and the overall mean motor summary T score (Table 4). Higher proportions of cases performed worse on the GP-DH (P = 0.037; Adj. P = 0.118, Table 5), but there were no significant differences in the proportions of cases and controls with impairment on GP-NDH or on overall motor function mean domain deficit score (Table 5).

**Table 4:** Comparisons of motor and SIP demographically-corrected T scores between controls and HIV+ patients.

Test	HIV− (N = 395)		HIV+ (N = 343)		Cohen’s d (95% CI)	P Value	P Value
	N	Mean (SD)	N	Mean (SD)			(adj.)
**Motor domain**							
Grooved pegboard–dominant hand	362	50.0 (10.0)	318	48.1 (10.6)	− 0.18 (− 0.33, − 0.03)	0.018	0.036
Grooved pegboard– non-dominant hand	362	50.0 (9.99)	318	49.0 (10.4)	− 0.10 (− 0.25, 0.05)	0.188	0.251
Motor summary score	362	50.0 (9.10)	318	48.6 (9.74)	− 0.15 (− 0.30, 0.001)	0.050	0.050
**SIP domain**							
WAIS-III digit symbol total	361	50.0 (9.98)	321	47.6 (9.62)	− 0.25 (− 0.40, − 0.10)	0.001	0.008
WAIS-III symbol search total	355	50.0 (9.98)	319	49.7 (11.0)	− 0.03 (− 0.18, 0.13)	0.745	0.745
Stroop color total	362	50.0 (10.0)	314	49.0 (11.1)	− 0.09 (− 0.24, 0.06)	0.233	0.266
Stroop words total	361	50.0 (9.99)	311	48.1 (10.3)	− 0.19 (− 0.34, − 0.04)	0.014	0.036
Trail making A time	363	50.0 (10.0)	321	48.1 (10.6)	− 0.18 (− 0.33, − 0.03)	0.017	0.036
Color trails 1 time	364	50.0 (10.0)	321	48.7 (9.58)	− 0.13 (− 0.28, 0.02)	0.084	0.134
SIP summary score	350	50.1 (6.62)	309	48.6 (6.95)	− 0.21 (− 0.37, − 0.06)	0.006	0.012

Cohen’s d compares HIV+ to HIV−; P value (adj.) = p value corrected for multiple testing; The higher the T score, the better NP performance is.

*SD* standard deviation, *CI* confidence interval, *SIP* speed of information processing, *WAIS-III* Wechsler Adult Intelligence Scale-*III.*

**Table 5:** Comparisons of proportions of impairment in motor and SIP domains between controls and HIV+ patients.

Test	HIV− (N = 395)		HIV+ (N = 343)		OR (95% CI)	P value	P value
	N	N impaired (%)	N	N impaired (%)			(adj.)
**Motor domain**							
Grooved Pegboard–dominant hand	362	60 (16.6%)	318	73 (23.0%)	1.50 (1.02, 2.19)	0.037	0.118
Grooved Pegboard–non-dominant hand	362	56 (15.5%)	318	59 (18.6%)	1.24 (0.83, 1.86)	0.285	0.326
Motor summary score	362	44 (12.2%)	318	46 (14.5%)	1.22 (0.78, 1.91)	0.375	0.375
**SIP domain**							
WAIS-III digit symbol total	361	44 (12.2%)	321	73 (22.7%)	2.12 (1.41, 3.19)	< 0.001	0.002
WAIS-III symbol search total	355	45 (12.7%)	319	52 (16.3%)	1.34 (0.87, 2.07)	0.182	0.243
Stroop color total	362	50 (13.8%)	314	60 (19.1%)	1.47 (0.98, 2.22)	0.064	0.118
Stroop words total	361	49 (13.6%)	311	58 (18.6%)	1.46 (0.96, 2.21)	0.074	0.118
Trail making A time	363	53 (14.6%)	321	65 (20.2%)	1.49 (1.00, 2.21)	0.052	0.118
Color trails 1 time	364	56 (15.4%)	321	56 (17.4%)	1.16 (0.78, 1.74)	0.467	0.467
SIP summary score	350	36 (10.3%)	309	64 (20.7%)	2.28 (1.47, 3.54)	< 0.001	0.001

Impaired, domain deficit score > 0.5 or individual test deficit score >  = 1.

*OR* odds ratio, compares HIV+ to HIV−, *P value (adj.)* p value corrected for multiple testing, *CI* confidence interval, *SIP* speed of information processing, *WAIS-III* Wechsler Adult Intelligence Scale-*III.*

#### SIP

Analysis of SIP data showed no significant difference in the WAIS-IIISS, Stroop Color Naming, or CTT1 T scores of cases and controls (Table 4). However, compared to controls, cases had significantly worse T scores on WAIS-IIIDS, Stroop Word Reading and TMT-A tests, and significantly lower overall mean SIP summary T scores (P = 0.006; Adj. P = 0.012, Table 4). Comparative analyses of the proportions with impairments in SIP show no significant differences in the proportions of cases and controls on WAIS-IIISS and CTT1 tests, and marginal differences on the Stroop Color Naming (P = 0.06; Adj. P = 0.12) and Stroop Word Reading (P = 0.07; Adj. P = 0.12). However, again, a significantly higher proportion of cases showed impairments on the WAIS-IIIDS (22.7%, P < 0.001; Adj. P = 0.002) and TMT-A (20.2%, P = 0.05; Adj. P = 0.12), compared respectively to 12.2% and 14.6% of controls (Table 5). The overall mean SIP domain deficit scores also showed that the proportion of cases with impairment in SIP (20.7%) was double that of controls (10.3%) (P < 0.001; Adj. P = 0.001, Table 5).

### Effects of ART on performance in NP tests of complex motor function and SIP

#### Motor

T scores on the GP-DH were not different between cases on treatment and those not on ART (*d*: 0.06, 95% CI − 0.17, 0.28; P = 0.619; Adj. P = 0.707). However, compared to cases not taking ART, those on treatment had significantly higher T scores on GP-NDH (*d*: 0.29, 95% CI 0.06, 0.51; P = 0.012; Adj. P = 0.048), with difference also in the overall mean motor summary T scores (*d*: 0.18, 95% CI − 0.04, 0.41; P = 0.107; Adj. P = 0.107).

#### SIP

Comparative analyses of cases on treatment (n = 171) and those who were not taking ART (n = 146) showed no effect of treatment on performance in WAIS-SS (*d*: 0.06, 95% CI − 0.16, 0.29; P = 0.578; Adj. P = 0.707), TMT-A (*d*: 0.01, 95% CI − 0.21, 0.24; P = 0.897; Adj. P = 0.897), or CTT1 (*d*: 0.13, 95% CI −0.09, 0.35; P = 0.259; Adj. P = 0.414) tests. However, cases on ART did show better performance on Stroop Word Reading (*d*: 0.23, 95% CI 0.01, 0.46; P = 0.043; Adj. P = 0.086) and on WAIS-IIIDS (*d*: 0.24, 95% CI 0.02, 0.47; P = 0.031; Adj. P = 0.083), but these differences became non-significant (at $\alpha$ = 0.05) when data were adjusted for multiple testing. Cases on ART had significantly better T scores on Stroop Color Naming (*d*: 0.33, 95% CI 0.10, 0.55; P = 0.004; Adj. P = 0.032), and the overall mean SIP summary T scores (*d*: 0.24, 95% CI 0.01, 0.46; P = 0.041; Adj. P = 0.082).

The types of ART regimens (cases on nevirapine- vs. cases on efavirenz-based ART; cases on zidovudine- vs. non-ZDV-based ART) and number of ART regimens (cases that had been on only one regimen vs. cases that had been on ≥ 2 ART regimens) had no effect on the mean motor or SIP summary T scores.

### Effects of current CD4+ cell counts on performance in NP tests of complex motor functions and SIP

#### Motor

Comparative analyses of cases with low CD4 (< 350 cells/μl, n = 116) and higher CD4 (350 cells/μl, n = 174) cell counts showed no influence of CD4 levels on T scores for GP-DH (*d*: − 0.04, 95% CI − 0.28, 0.2; P = 0.731; Adj. P = 0.793), GP-NDH (*d*: 0.14, 95% CI − 0.09, 0.38; P = 0.234; Adj. P = 0.468), or the mean motor summary T scores (*d*: 0.05, 95% CI − 0.18, 0.29; P = 0.652; Adj. P = 0.652).

#### SIP

There were no significant differences in T scores of cases with low and higher CD4 counts for WAIS-IIISS (*d*: 0.11, 95% CI − 0.13, 0.34; P = 0.373; Adj. P = 0.597), Stroop Color Naming (*d*: 0.04, 95% CI − 0.2, 0.28; P = 0.729; Adj. P = 0.793), or Stroop Word Reading (*d*: 0.03, 95% CI − 0.21, 0.27; P = 0.793; Adj. P = 0.793). However, cases with higher CD4 counts had significantly higher T scores on WAIS-IIIDS (*d*: 0.29, 95% CI 0.05, 0.53; P = 0.017; Adj. P = 0.136), and marginally higher T scores on TMT-A (*d*: 0.21, 95% CI − 0.03, 0.44; P = 0.083; Adj. P = 0.253), CTT1 (*d*: 0.2, 95% CI − 0.04, 0.44; P = 0.095; Adj. P = 0.253), and marginally higher overall mean SIP summary T scores (*d*: 0.22, 95% CI − 0.03, 0.46; P = 0.078; Adj. P = 0.156).

### Effects of viremia and viral subtypes on performance in NP tests of complex motor functions and SIP

Comparative analyses of cases with controlled viremia (undetectable VL) and cases with detectable VL showed no effect of viremia on cases’ T scores for GP-DH, GP-NDH, or the mean motor summary T scores. Similarly, comparative analyses showed no effect of systemic viremia on T scores for WAIS-IIIDS, WAIS-IIISS, Stroop Colors and Words, TMT-A, or CTT1, and no effect of viremia on the overall mean SIP summary Tscores.

To determine if successful treatment influence NP performance, we performed comparative analyses of cases not on ART (n = 127), cases on ART that had detectable VL (n = 36), and cases on ART that had undetectable VL (n = 128). Pairwise comparisons of mean T scores showed that compared to cases on ART that had undetectable VL, cases not taking ART had significantly lower (poorer) T scores on GP-NDH (*d*: − 0.33, 95% CI − 0.58, − 0.08, P = 0.03) and Stroop Color Naming (*d*: − 0.28, 95% CI − 0.37, − 0.11, P = 0.014); with T scores of cases not on ART lower than T scores of cases on ART that had detectable VL, and T scores for this latter group lower than T scores of cases on ART that had undetectable VL.

Additional analyses of cases infected with HIV−1 CRF02_AG (n = 88) compared to cases infected with other subtypes (n = 63) showed no significant effect of viral subtype on mean T scores for tests of motor function or SIP between the two groups.

## Discussion

There is limited knowledge on the neurocognitive effects of HIV infection in SSA, and accurate assessment of the neuropsychological effects of HIV/AIDS requires population-appropriate norms. In fact, a recent meta-analysis showed that normative data from different countries and cultures are frequently not equivalent^44^, further underscoring the need for population- and culture-appropriate norms to avoid errors in the diagnosis of NCI, as well as the need to adjust NP analyses for demographic factors that may also differ across populations. The current study provides Cameroonian normative standards for commonly used measures of SIP and complex motor functions.

The GP is a frequently used test of fine motor function that is part of the World Health Organization NP test battery used to assess neurological health and function across diverse cultural contexts^45,46^. It assesses psychomotor functions such as manual dexterity, upper-limb motor speed, and visuo-motor coordination^47^. Performance on the GP test is used to assess motor impairment^48^ and correlates with function in other cognitive domains such as memory, attention, SIP, and executive function^49–51^. Diseases that affect the CNS such as multiple sclerosis^51,52^, Parkinson’s disease^53–55^, and HAND^56^ are associated with prolonged time in completing the GP test. In the current study, HIV effects were observed only for GP-DH, which drove the overall HIV effect on the motor summary T score. However, there was no significant difference in the proportions of cases and controls with impairment in the motor domain, suggesting either that the GP test is not very sensitive for detecting HIV-associated deficits in fine motor function in this relatively young population, or that complex motor function is not commonly affected in Cameroonians with HAND.

The tests used to assess SIP in this study included the WAIS-IIIDS and WAIS-IIISS, Stroop Word Reading, Stroop Color Naming, TMT-A, and CTT1. The WAIS-IIISS, Stroop Color Naming, and CCT1 did not show evidence of HIV effects, but significant HIV effects were observed with the WAIS-IIIDS, Stroop Word Reading, TMT-A, and the overall SIP summary T scores, with the proportion of HIV+ cases that had impairment in SIP (20.7%) double that of controls (10.3%). The TMT-A measures speed of visuomotor and cognitive tracking^57^. The Digit Symbol subtest of WAIS-III primarily measures mental processing speed and clerical efficiency^39,58^. Poor performance on these tests correlates with deficits in speed of information processing and visuomotor response^59^, as well as deficits in working memory^60^. The current results demonstrate that HIV infection in Cameroon is associated with significant deficits in SIP and confirm our previous pilot data^61^, as well as studies in other settings showing impairment in SIP among PLWH^56,62,63^.

It is well known that performance on NP tests raw scores is influenced by demographic factors such as age, education, and gender; as well as by race/ethnicity and cultural backgrounds^33,35^. In the current study, the control/seronegative group showed age and education effects on the GP test, and also significant effects of age and education on tests of SIP, with younger and more educated controls performing significantly better on these tests, compared to older and less educated controls. However, all these demographic effects were eliminated in corrected T scores. Although there was no effect of age or education on tests of motor function T scores among cases, male cases had significantly better T scores than females cases on the GP-NDH. However, there was no gender effect on cases’ overall motor summary T scores. Performance of cases on several individual tests of SIP and the overall SIP summary T scores also showed gender, age, and education effects, even after all such effects found in the healthy controls were corrected with conversion of raw scores into T scores; with females, older, and less educated cases performing significantly worse than males, younger, and more educated cases. These results agree with previous findings in both high-income^33,35,64^ and resources-limited^10,65,66^ settings, suggesting increased vulnerability to HIV effects being associated with demographic characteristics (older age, lower education, female gender) that tend to influence poorer absolute levels of performance (raw scores) on many of these tests. This may be explained by the concept of “cognitive reserve” where weaker premorbid abilities could make some subjects more vulnerable to illness or injury affecting the brain^67,68^.

Studies of PLWH in other settings, including in SSA^69^, Europe^70^ and the US^64,71^ also showed sex effects. Compared to HIV-infected males, infected females had higher prevalence of neurocognitive impairment^64,69,71,72^, with 1.5–2.17 times higher odds of HAND^71,72^, whereas the prevalence of impairment was similar for seronegative males and females^71,72^. Domain-specific studies also showed that psychomotor function was preferentially impaired in HIV+ females^70^; infected women had significantly lower T scores on TMT-A and GP-NDH, and longitudinal analyses showed that this sex difference remained over time^64^. The mechanisms responsible for sex differences in NP tests scores have not been elucidated. It has been suggested that social factors such as lower education for women may contribute to poor performance in NP tests. However, analyses were controlled for education and “normal” sex effects on NP tests scores were not seen among seronegative women, compared to seronegative men^71^. Biological/hormonal differences may also play a role. It has been suggested that women are more susceptible to the damaging effects of HIV on neurocognitive function because fluctuations in hormonal activities influence cognitive performance^73^, and brain regions often affected by HIV such as the striatum, prefrontal cortex and hippocampus have high concentrations of estrogen receptors^74–77^. It is not known whether following HIV infection, covariates such as poverty and other life stressors also contribute to these neurocognitive differences.

In our current study, ART had no effect on GP-NDH, WAIS-IIISS, TMT-A, or CCT1 T scores. However, ART use was associated with significantly higher T scores on GP-NDH and the overall motor summary score, as well as significantly higher T scores on WAIS-IIIDS and Stroop Color Naming and the overall SIP summary T score. Although these cross-sectional findings cannot establish causality, they do suggest that ART may improve motor function and SIP in HIV+ Cameroonians, similar to findings elsewhere. In fact, longitudinal studies of PLWH in other settings showed significant improvement in subjects’ scores on the GP-NDH, TMT, and Symbol Digit modalities tests following ART^6^. Improvement in GP scores and psychomotor function was observed in cases that had poor (low) GP scores at baseline (time of ART initiation), as well as in cases that had better GP scores at ART initiation^6–8^. In addition to improving performance in tests of fine motor functions and speed of mental processing, ART use is also associated with improved performance in NP tests assessing concentration, memory and mental flexibility^5^; and treatment failure is associated with poor performance on NP tests^9^. Despite these positive effects of ART on performance on some NP tests and the fact that ART use is associated with decreased prevalence of HAD, the overall prevalence of HAND in the current ART era remains high^5,12,13^.

Studies of diverse populations of PLWH in different settings showed that higher nadir CD4 counts were associated with reduced likelihood of HAND, whereas low nadir CD4 counts predicted cerebral atrophy^78^ and neurocognitive impairment^79–84^. In the current ART era there have been conflicting evidence as to whether there is a link between current immunosuppression and risk of neurocognitive impairment. Some studies showed no link between current CD4 levels and performance on NP tests^84–87^ whereas others showed that current low CD4 counts were associated with neurocognitive deficits^88–92^, including poor performance on tests of psychomotor speed^93^; and higher CD4 counts were associated with lower risk of HAND^72^. In longitudinal studies assessing ART effects on the immune system and HAND, the strongest improvements in neurocognitive and neurological functioning correlated with increased CD4 counts and were associated with increased treatment duration^8,94^. In our current study, better immune function also was associated with better SIP, with higher T scores on WAIS-IIIDS, TMT-A, and CTT1 for cases having higher CD4 compared to cases with lower CD4 counts.

The observed HIV-associated impairments in SIP and psychomotor functions in HIV-infected Cameroonians can have both biological and functional implications. In fact, other studies of HIV+ adults showed an association between cognitive function and brain metabolism, with a correlation between performance on TMT, WAIS-IIIDS, and GP-NDH and levels of the brain metabolites glutamine, glutamate, and *N*-acetyl aspartate^95^. Poor performance on tests of psychomotor function significantly correlated with increased inflammation, including increased blood interleukin-6 levels^96^, and increased likelihood of non-adherence to treatment^97^. Our future studies will investigate whether there is a link between NP test scores and adherence to ART in Cameroon, or a link between NP test scores and specific blood biomarkers.

In summary, the current study provides new Cameroonian adult normative standards for NP tests of psychomotor functions and SIP, including regression-based formulae for calculating T scores adjusted for age, education, and gender. These normative values will be useful for future studies of the neurobehavioral effects of diseases affecting the CNS in this country. Limitations of our current study include the fact that most of the subjects came from Yaoundé and its suburban neighborhoods. However, the 3 million inhabitants of Yaoundé, the Cameroon capital city, actually come from various parts of the country and include people from all Cameroon tribal and ethnic groups. It is well known that throughout SSA, over two-thirds of PLWH are females^98^, and in our current study 78% of cases and 65% of controls were females. Thus, limitations of the current study also included group differences in gender distribution, age, education. However, the “normal” effects of these demographic factors on NP performance were eliminated or strongly attenuated by the T score conversions presented here.

## Methods

### Psychometric instruments

The NP tests used in this study included: (1) tests assessing SIP [(a) WAIS-IIIDS and WAIS-IIISS subtests^39^, (b) Stroop Color Naming and Word Reading tests^40^, (c) TMT-A^41^, and (d) CTT1^42^]; and (2) tests of complex motor function (GP-DH and GP-NDH)^43^. Administration of NP tests and scoring were performed according to published standardized procedures and protocols outlined in test developers’ manuals. A brief description of these NP tests is provided below.

#### WAIS-III digit symbol^39^

For WAIS-IIIDS, the subject is presented with numbers associated with specific symbols (total of 9 digit-symbol pairs), then asked to match symbols to numbers on a sheet of paper as fast as possible (over a xaximum time of 120 s) and according to the digit-symbol key. The raw score consists of the number of correct symbols matched. WAIS-IIIDS measures processing speed, visual perception, attention, concentration, visual-motor coordination, motor and mental speed.

#### WAIS-III symbol search^39^

For WAIS-IIISS, the subject is shown a target symbol and then asked to scan search the target symbol in a group that includes a set of distractor symbols, and mark whether the target symbol is present or not. The subject has to respond to as many items as possible over a maximum of 120 s. The raw score consists of the number of correct responses minus the number of incorrect responses, and the maximum total score is 60. WAIS-IIISS assess SIP, perception, visual recognition and visual working memory.

#### Stroop color naming and word reading speed^40^

The Stroop test was administered over 45 s as we previously described^30^, with scores consisting of the total number of words read and total number of colors correctly identified and named. This test measures cognitive processing, mental speed and mental control.

#### Trail making test part-A time (TMT-A)^41^ and color trails-1 time (CTT1) ^42^

For TMT-A, the respondent had to rapidly draw a line linking numbers in sequence; the score consisted of time (seconds) taken to complete the task. For CTT1, the respondent used a pencil to rapidly connect circles (on a sheet of paper) numbered 1–25 in sequence. A stopwatch was used to record each trail completion time. TMT-A and CTT1 tests produce measures of attention, visual searching, visuomotor tracking, and psychomotor speed.

#### Grooved pegboard test^43^

This is a manual dexterity test that assesses fine motor functions and requires complex visual-motor coordination. The test unit consists of a peg tray and a board of 25 holes with randomly positioned slots such that insertion of the peg requires a rotation of the peg key to match the groove of the peg with the groove of the board. The respondent has to put the pegs into the holes as fast as possible and in order, first using the dominant hand, and then repeating the test using non-dominant hand. A stopwatch was used to record the time (seconds) taken to complete each trial and the number of pegs dropped recorded.

### Adaptation of NP tests and study population

The NP tests and test instructions were translated into French, back-translated, standardized and pilot-tested in Cameroon and quality assurance reviews were done on randomly selected data files as previously described^61^. These tests were part of a larger international NP test battery assembled by the University of California San Diego HIV Neurobehavioral Research Center (HNRC)^61^. This battery includes 19 NP tests assessing 7 cognitive domains, and has been successfully used to detect HAND in developed and resource-limited countries, including countries in North America^15,56^, South America^99^, Asia^100–102^, and in SSA^19,61,103–105^. Because combining normative data for all 19 tests with data and discussion regarding viral factors, ART, immunological data and their effects on neurocognitive performance, would be excessive for a single manuscript, this report focuses on SIP and complex motor functions. All study participants spoke French and all tests were administered in French. Subject recruitment, inclusion and exclusion criteria were done as previously described^61^ and subject characteristics are summarized in Table 1. We recruited a total of 683 subjects, including 363 healthy HIV− controls and 320 HIV+ cases.

### Norming procedure and analyses of NP data

Data norming was performed according to published procedures^30,106,107^. Briefly, for each NP test, raw scores were standardized and converted into normalized scaled scores; and scaled scores fitted to a multivariable fractional polynomial (MFP) model^106^, using R package mfp (https://cran.r-project.org), to convert into T scores corrected for age, education, and gender. The formulas developed using the normative group (HIV− controls) were then used to calculate T scores of the HIV+ group; T scores on the individual test measures were then used to calculate deficit scores for the tests and the SIP and motor domains^56^.

### Laboratory analyses

Following NP testing, urine samples were collected to test for substance use; and blood samples were collected for HIV serology, CD4 counts, and VL. Two different commercially available tests (rapid immunochromatographic HIV-1/2 test and the Murex HIV antigen/antibody Combination ELISA, Abbott Diagnostics, Chicago, IL, USA) were used per manufacturer’s instructions to determine HIV serology. CD4 T-lymphocyte levels were quantified by flow cytometry, VL by reverse transcription polymerase chain reaction (RT-PCR), and viral genes amplified and sequenced as previously described^37,38^.

### Statistical analyses

Comparative analyses of cases’ and controls’ demographic data were performed using the Student’s *t* tests (for continuous variables) and Fisher's exact test (for binary variables). For both HIV− controls and HIV+ groups, univariable analysis and multivariable linear regression were employed to determine the association between demographic factors (age, gender, and education) and T scores for SIP (WAIS-IIIDS, WAIS-IIISS, Stroop Color and Word tests, CTT1 and TMT-A) and motor function (GP-DH and GP-NDH). Analyses of T scores and prevalence of impairment were performed respectively using linear and logistic regression models. Logistic regressions were used for comparative analyses of the proportions of impairments in SIP and motor function between cases and controls: impaired if domain mean deficit score > 0.5 or individual test deficit score ≥ 1. Further analyses of cases were performed to determine the effects of treatment status (untreated and on ART), successful treatment (on ART with undetectable VL, on ART with detectable VL, and untreated), CD4 + T-cells counts (< 350 and ≥ 350 cells/µl), and VL (undetectable and detectable) on T scores. In addition, the P values for the analyses of individual tests (k = 8) and for the analyses of individual domain scores (k = 2) were corrected for multiple testing using false discovery rate method that takes into account the number (k) of P values to be corrected and the magnitude of each uncorrected P value. These adjusted P values are labeled as “Adj. P”.

### Ethical approval and study participants

This study was approved by the University of Nebraska Medical Center Institutional Review Board (IRB #307-06-FB) and the Cameroon National Ethics Committee (Ethical Clearance Authorization #146/CNE/SE/2012); and conducted in compliance with the Helsinki Declaration. Subjects were recruited from four different hospitals and health care centers in Yaoundé, Cameroon. All subjects ≥ 18 years old who met no exclusion criteria (i.e., no history of psychiatric or CNS diseases, traumatic brain injury, no current fever or non-HIV systemic illness, and no current drug intoxication)^61^ were invited to participate in the study. Written informed consent was obtained from all participants.

## Data Availability

Nucleotide sequences for clinical isolates reported in this study are available in the NCBI database; Genbank accession numbers included in our previous publicationsy^37,38^.
